# Evidence that digital game players neglect age classification systems when deciding which games to play

**DOI:** 10.1371/journal.pone.0263560

**Published:** 2022-02-22

**Authors:** Ross Hollett, Sian Tomkinson, Sam Illingworth, Brad Power, Tauel Harper

**Affiliations:** 1 Department of Psychology, School of Arts and the Humanities, Edith Cowan University, Perth, Western Australia, Australia; 2 Media and Communication, School of Social Sciences, Faculty of Arts, Business, Law and Education, The University of Western Australia, Perth, Western Australia, Australia; 3 Department of Learning and Teaching Enhancement, Edinburgh Napier University, Scotland, United Kingdom; 4 College of Arts, Business, Law and Social Science, Murdoch University, Perth, Western Australia, Australia; University of Milan, ITALY

## Abstract

This article considers players’ experiences seeking out new games to play, and their use of the Australian National Classification Scheme in doing so. The global video game industry is booming, with hundreds of games being released each month across numerous platforms. As a result, players have an unprecedented number of games available when choosing what games to purchase. However, a number of confounding issues around the emergent content of games and the subjective nature of game reviewing makes it difficult to relate what kinds of experiences a given game will facilitate. In this study, we surveyed game players in order to find their game platform and acquisition preferences; strategies and experiences when choosing games; and attitudes towards classification systems. Our findings suggest that players find it difficult to choose what games to purchase, and that existing classification systems are mostly only beneficial when choosing games for minors.

## Introduction

It is well-known that the video game industry is growing rapidly and has surpassed the film industry on numerous fronts. By the end of 2020 there were 2.7 billion players worldwide [1], and the global games market generated $174.9 billion USD, about half of this being generated from mobile games [2]. There are huge numbers of games being published with over ten thousand released on the popular video game digital distribution service ‘Steam’ alone in 2020, or around 600 to a thousand per month [3]. With such a saturated and competitive market, it is often difficult and expensive for game developers to reach the right market of players, and for players to easily find games which suit their needs [4]. Given the sheer number of games available, paired with the subjective nature of game experiences [5] and misleading advertising practices common within the industry, the complex task of choosing a game to play has become inherently challenging. Consequently, the aim of this study was to better understand the consumer experience of choosing digital games, with a particular focus on evaluating the utility of National Media Classification Systems, such as Australia’s National Classification Scheme.

## Background

The pressure for success has led to some misleading marketing tactics in the game industry. There have been several high-profile cases of deceptive marketing practices where game companies have failed to deliver on content and features (e.g., smooth performance) promised during game development. In some cases, these high-profile games are rushed into market with considerable flaws (i.e., “bugs”) which further undermines consumer trust, such as *No Man’s Sky* [6], and *Cyperpunk 2077*. Further, the need for paid sponsorships have resulted in a lack of trust in games journalism, influencer reviews, and even game developers themselves [7]. For example, in 2007 GameSpot’s advertising deal with Eidos Interactive led to a journalist being fired for providing a poor review of their game, *Kane & Lynch* [8]. Similarly, while rooted in toxic gamer culture, the Gamergate incident of 2014 actually congealed around a common belief in unethical reviewing practices within the industry [9]. In 2016, Warner Bros. settled charges from the Federal Trade Commission, which asserted that they had failed “to adequately disclose that it paid online ‘influencers,’…thousands of dollars to post positive gameplay videos [of *Shadow of Mordor*] on YouTube and social media” [10]. Because of these issues, players often lack trust in reviews as a guide for game choice, particularly when it comes to big budget AAA releases. In this case National Classification Systems may help players understand the content of games because they are free from commercial bias.

### Benefits and limitations of national classification systems

Media classification systems, such as the IARC (International Age Rating Coalition), PEGI (Pan European Game Information) and ESRB (Entertainment Software Ratings Board) operate as a form of media content regulation in various jurisdictions worldwide [11]. Classification systems have been developed as governments have, in general, progressed from a censorship model to one where all material are provided with classifications and only exceptional material is censored [12]. Classification systems contain age and/or maturity-based levels, indicating the minimum recommended age one should be to engage with a piece of media or advising the parent/guardian that they should provide guidance to their child depending on their maturity level [13]. They take elements such as violence, sex, and drug use into account when determining the appropriate audience age range for any given piece of media. They vary country-by-country according to political, cultural, and religious influences [13–16], and there have been calls to design a worldwide system [14], although it would be significantly challenging to establish a common understanding of what age-appropriate means [14]. While classification systems’ primary role is to govern “what pleasures, knowledge and experiences are deemed appropriate for minors” [17] and, more broadly, protect individuals from material they find offensive [18], these classifications also provide commercially unbiased summaries of game content.

Australia’s ‘National Classification Scheme’, is overseen by the Commonwealth (federal) Government as well as state and territory governments. It involves the ‘National Classification Code’, which was established in May 2005 and was approved by all Commonwealth, State and Territory Censorship ministers [18]. This document outlines the purpose of the code and the way that media are to be classified. In this code, publications, films, and computer games each have a different system. Computer games can be rated the following way [18]:

- RC: Refused Classification.
- R 18+: “unsuitable for viewing or playing by a minor” (introduced in 2013)
- MA 15+: “Computer games … that depict, express or otherwise deal with sex, violence or coarse language in such a manner as to be unsuitable for viewing or playing by persons under 15”.
- M: “Computer games…that cannot be recommended for viewing or playing by persons who are under 15”.
- PG: “Computer games…that cannot be recommended for viewing or playing by persons who are under 15 without the guidance of their parents or guardians”.
- G: “All other computer games”.

“The national scheme is implemented through the Commonwealth Classification (Publications, Films and Computer Games) Act 1995”, which is in turn “supplemented by a number of regulations, determinations and legislative instruments” [19]. The Commonwealth manages the Classification Board and Classification Review Board [19–21], which decide what classification a given piece of media should receive, and manages appeals to that decision. There are also two classification tools available which produce classification decisions via a questionnaire or computer program. These are “The Global Rating Tool for the classification of mobile and online games on participating storefronts”, and “The Netflix Classification Tool” [19]. States and territories “make laws about how films, computer games, and publications can be distributed, shown and advertised” [19].

The role of classification systems as cultural arbiter is often problematic. For instance the Australian Classification Board is viewed as quite harsh towards video games [22], and there is some confusion regarding the M and MA15+ ratings [13]. Some games that were refused classification in Australia prior to the introduction of the R 18+ rating were available in other countries according to their classification systems, or were reevaluated as MA15+, some with adjustments, others without [13]. Even after the introduction of the R 18+ rating in 2013, numerous games are still refused classification, typically due to violence or drug use [23]. Indeed, Australia and Singapore are the only countries where a game can be banned if its content cannot be accommodated by the rating system [15]. Such concern is evident in other nations’ systems, with the US’s ESRB focusing on protecting young children from violent and sexual content, and the German Unterhaltungssoftware Selbstkontrolle regulating violent shooter games more strictly than others [16, 24]. In 2020 the Australian Government announced a review of the National Classification Scheme, and many of the invited submissions sent from public bodies, corporations, and game developers, among others, alluded to the complex and at times opaque nature of the process [25].

### Alternatives to national classification systems

Our research indicates that players engage in numerous alternative methods to choose games. It is understood that players find new games through recommendations from family and friends; YouTubers (or influencers); social media; gaming websites; gaming magazines; TV advertisements; game developer websites; and expos, all carrying varying levels of trust (as noted above in the case of journalism) [26]. Other approaches include recommendation and tagging systems (such as that used on Steam) [27], and browsing forums such as reddit.

There is much research on the reasons for why players choose certain games in regard to personality traits and game genres [28–30], but little on the actual seeking out, decision-making, and purchasing process.

Considering the many channels through which players find games to play, the time required to explore these channels, and the inherent difficulty in describing and differentiating game experiences as described above, it is important to understand attitudes towards the game selection process. Specifically, we feel this understanding will benefit not only players, but also game creators/publishers, by justifying the development of more informative systems for describing game content.

### The present study

Given the wide variety of strategies available to players to evaluate game suitability and the inherent difficulty of selecting games from the immense volume of available content, we sought to better understand how players make game purchasing decisions and their attitudes towards the experience. We used both quantitative and qualitative methods to explore player experiences so that fixed response categories could be further contextualised with more detailed descriptions. We chose to address the following research questions:

1. What are the leading strategies used by players to choose games to play?
2. Do players positively or negatively appraise the game selection process?
3. Do players regard the game selection process as difficult?
4. What are players’ attitudes towards the Australian National Classification Scheme?
5. Would players be supportive of a more informative classification system?

## Method

### Participants

Participants were 210 digital game players (59% female) who reported playing games for an average of 5.70 hours per week (*SD* = 5.60). Players were aged between 17 and 70 years (*M* = 31.45, *SD* = 12.22) and mostly identified as Australian (68%).

### Measures

#### Game platform and acquisition preferences

Participants were requested to select which platform(s) they use to play digital games from a list of popular options (PC, mobile/tablet, PlayStation, etc.). Participants were also requested to select how they access (by purchase or for free) digital games from a list of popular options (online retailers, app stores, bricks-and-mortar stores, etc.). Finally, participants were requested to estimate how much money they spend (in Australian dollars) on digital games (including subscriptions) each year by selecting from several categories which ranged from *$0* to *More than $1000*.

#### Game decision strategies and experiences

Participants were requested to estimate on a 5-point scale (anchored with *Never* to *Always*) how often they make use of popular game decision strategies (e.g., Australian National Classification Scheme, game reviews, etc.). Participants were also given the option to select “other” and enter a text response to capture any other strategies not offered by the provided list. Participants were also requested to rate their experience of choosing a digital game on a 5-point scale (anchored with *Strongly disagree* to *Strongly agree*) for six different positive and negative descriptors (enjoyable, time consuming, frustrating, confusing, boring, satisfying). A further item was included using the same 5-point scale to measure whether participants found it difficult to determine whether a digital game would meet their needs before playing it.

#### Attitudes towards classification systems

Participants were requested to estimate on a 5-point scale (anchored with *Never* to *Always*) how often they pay attention to classifications when choosing a game for themselves and (if applicable) for a child (under 16). Participants were also asked two questions on 5-point scale (anchored with *Strongly disagree* to *Strongly agree*) regarding their agreement that the Australian Government classification system already adequately (1) assists them in deciding which games to play and (2) describes the content of digital games in Australia. A further question was asked on the same 5-point scale which sought to measure whether participants would be supportive of a more informative classification system that would assist them in choosing a game that meets their needs. Participants were also given a text option to add further detail explaining their attitudes towards classification systems.

### Procedure

The survey link was distributed via a snowballing method through professional and social networks, with an open invitation to pass the survey along to other interested parties. The survey was at various points also hosted on our research lab’s website and on our Facebook page. A randomly drawn prize of two AU$50 gift vouchers was also used in messaging to incentivise survey completion. All participants provided informed consent for their anonymous responses to be included in this study by completing a consent form before beginning the questionnaire. These procedures were approved by the lead and co-author’s university ethics committees.

## Results

### Game platform and acquisition preferences

**Fig 1:**
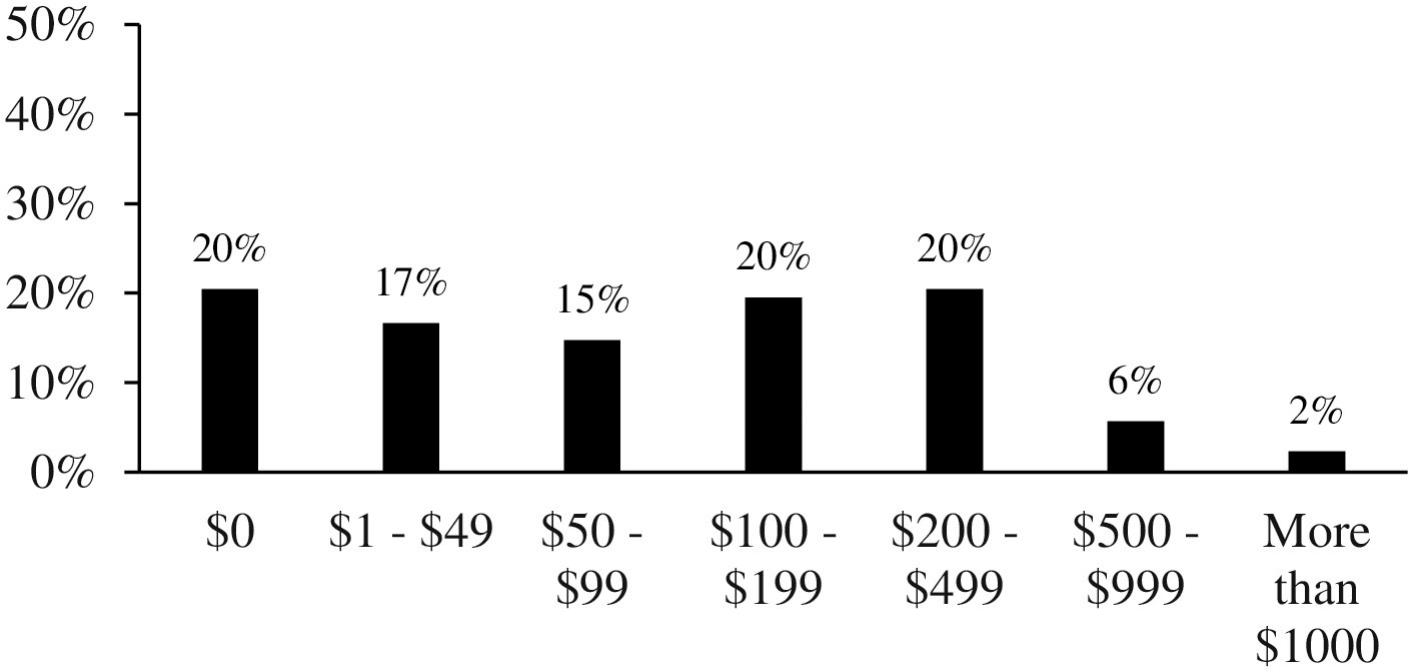
Response distribution for annual spend on digital games.

Most players reported using a personal computer/laptop to play digital games (68%), followed by mobile/tablet (59%), PlayStation (33%), Nintendo Switch (27%), Xbox (18%), Nintendo Wii (8%), and Nintendo DS (8%). Most players reported downloading purchased games from an online retailer (e.g., Steam) to a PC or console (58%), followed by downloading free games to a phone or tablet from an app store (57%), downloading free games from an online retailer (e.g., Steam) to a PC or console (43%), purchasing a hardcopy from a bricks-and-mortar retail outlet (34%), downloading purchased games to a phone or tablet (22%), sharing with a friend (19%), purchasing a hardcopy from an online retailer (13%), peer to peer sharing/torrenting (8%), online streaming (7%), and purchasing a digital copy from a bricks-and-mortar retail outlet (4%). We have also reported the response distribution for annual spending of players on digital games (including subscriptions) in Fig 1, with around half of players spending less than $100 per annum on digital games.

### Game decision strategies and experiences

**Fig 2:**
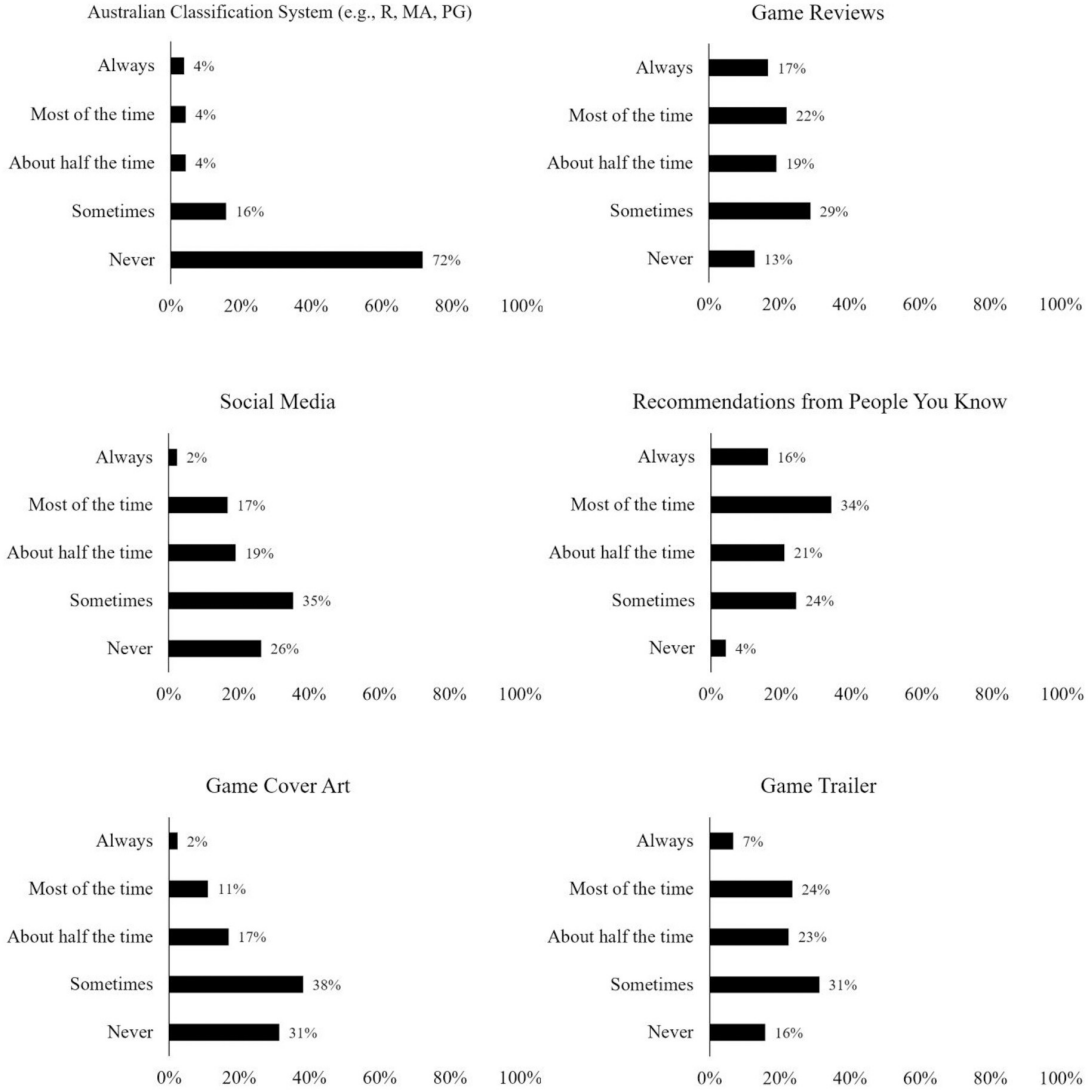
Response distributions for game decision strategies.

Fig 2 illustrates the response distributions for the strategies that players reported using to make decisions when seeking a game which meets their needs. Note that most distributions showed reasonable spread except for the use of the Australian Classification System of which more than 70% participants reported never using. Specifically, the most popular strategy was recommendations from people participants knew, followed by game reviews, game trailers, social media, game cover art, and the Australian Classification System.

As 14% of participants selected “other”, their text responses were analysed and were found to suggest a number of novel game decision strategies. For instance, 24% of these text responses identified communities of play as influencing their decisions—these communities included personal networks, community media (such as reddit, Twitch and YouTube), or Steam community ratings. A further 20% listed the game description, and factors such as the games developer, the game’s franchise and the price each accounted for 12% of written responses. Other written explanations for factors that influenced game choice included “game mechanics” (8%) and “time”, “past experience” and trying a torrented version of the game before buying (4% each). Numerous written responses alluded to issues of trust, noting a difference between critic and audience reviews. Respondents were conscious of whether a review is paid, credible, of quality, and is trustworthy.

**Fig 3:**
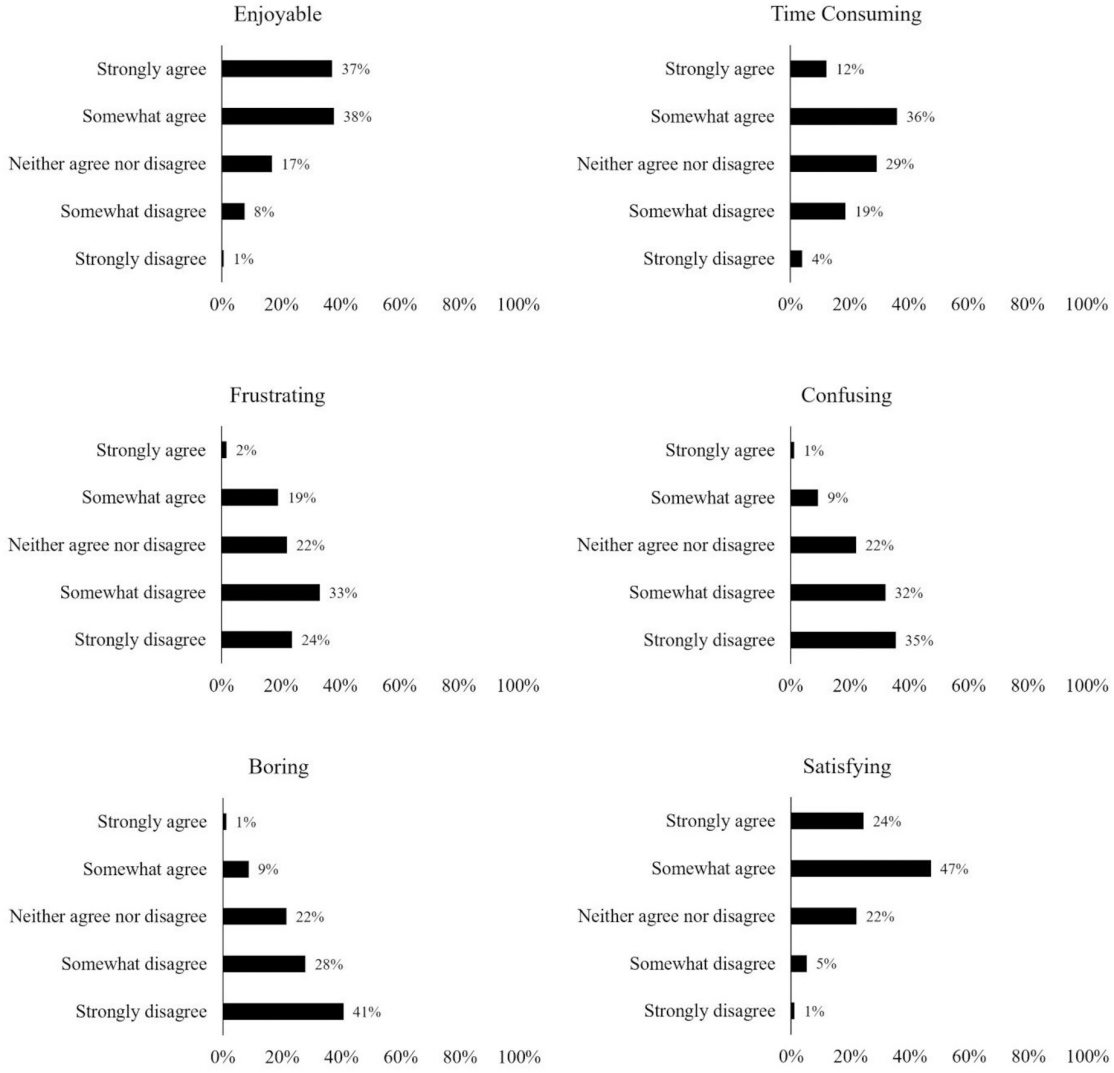
Response distributions for the game decision experience.

Fig 3 illustrates the response distributions from participants regarding their experience of the game decision process when choosing a game that meets their needs (note that these data only included participants who purchase games for themselves, *N* = 172).

Overall, the game decision process appeared to be positive with most participants agreeing that the experience is enjoyable and satisfying. Furthermore, large proportions of participants disagreed that the process is frustrating, confusing, or boring. However, a large proportion (48%) of participants agreed that the process is time consuming.

**Fig 4:**
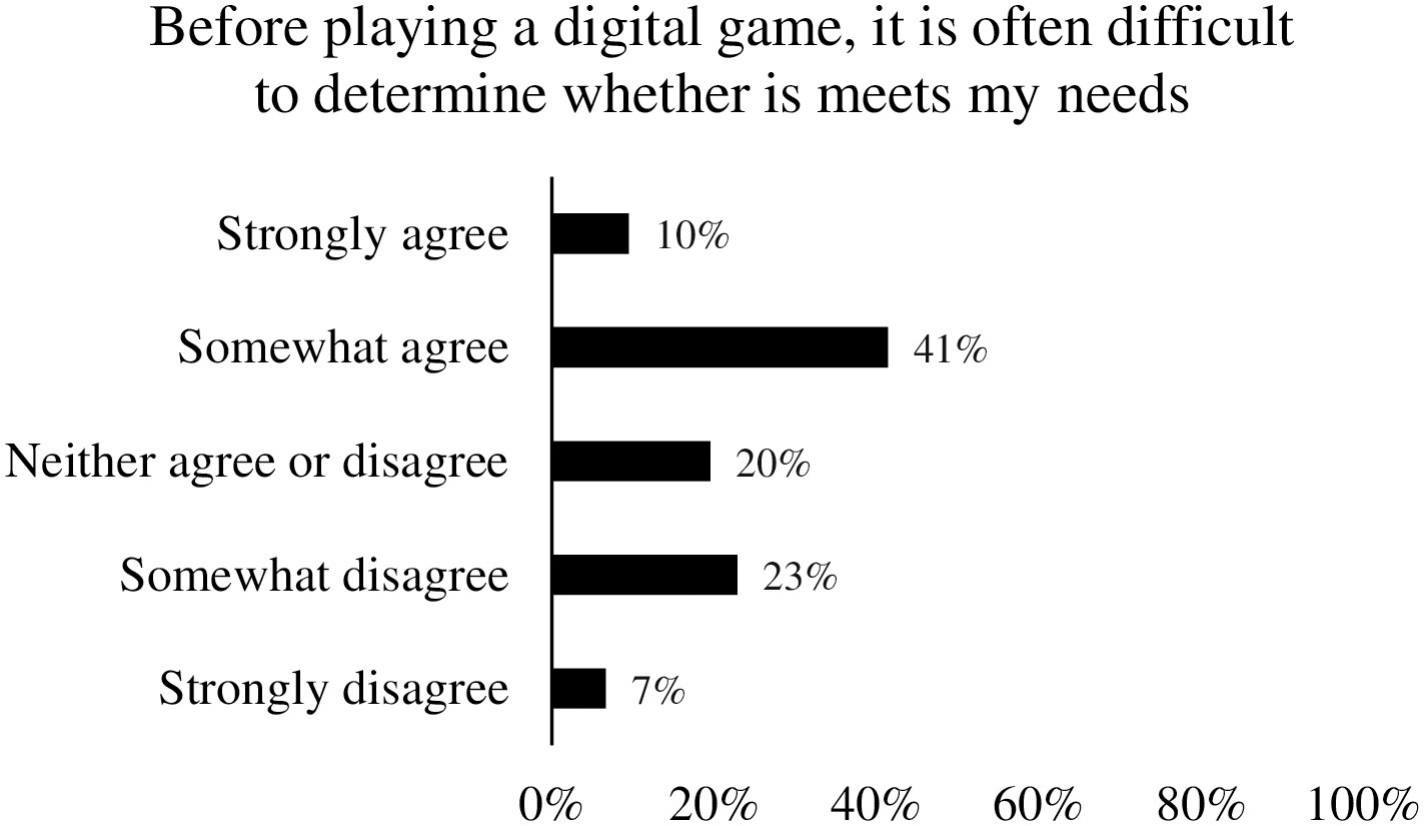
Response distribution for difficulty determining game suitability.

We also found that around half of participants agreed that it is often difficult to determine whether a game will meet their needs before playing it (see Fig 4).

### Attitudes towards classification systems

**Fig 5:**
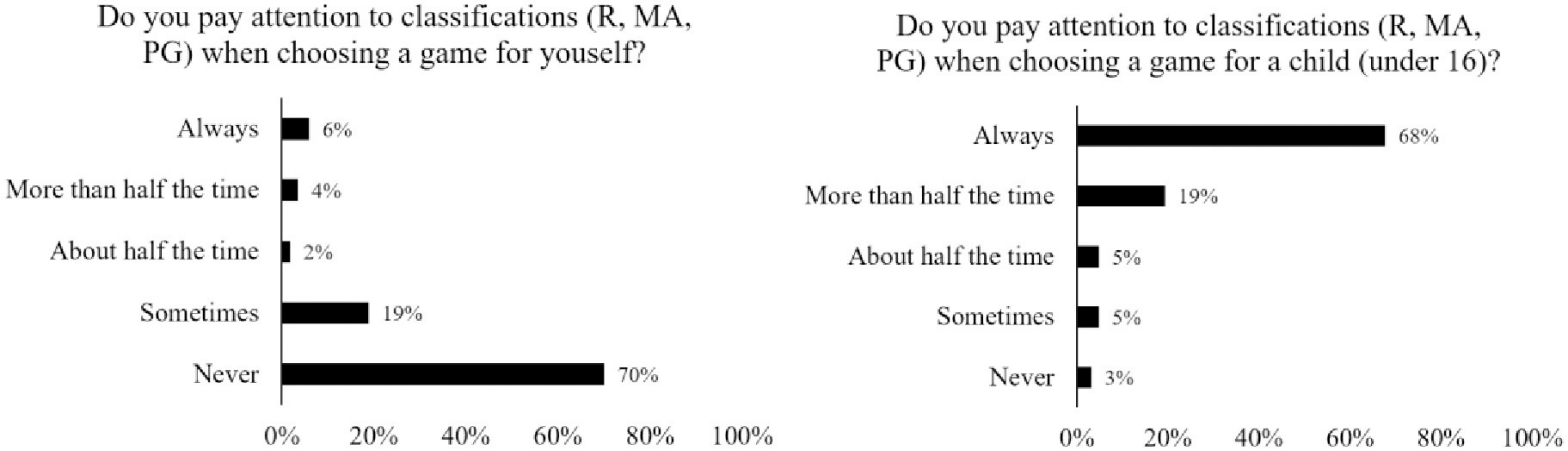
Response distributions regarding attention to classifications when choosing for the self and for children.

Fig 5 illustrates the attention participants estimated giving to classifications when choosing a game for themselves or a child. Note that the response data here is separated based on whether people buy for themselves (*N* = 170) or/and buy for children (*N* = 62). Importantly, it is clear that classifications are largely disregarded by participants who were choosing a game for themselves but is useful for participants who are choosing games for children.

**Fig 6:**
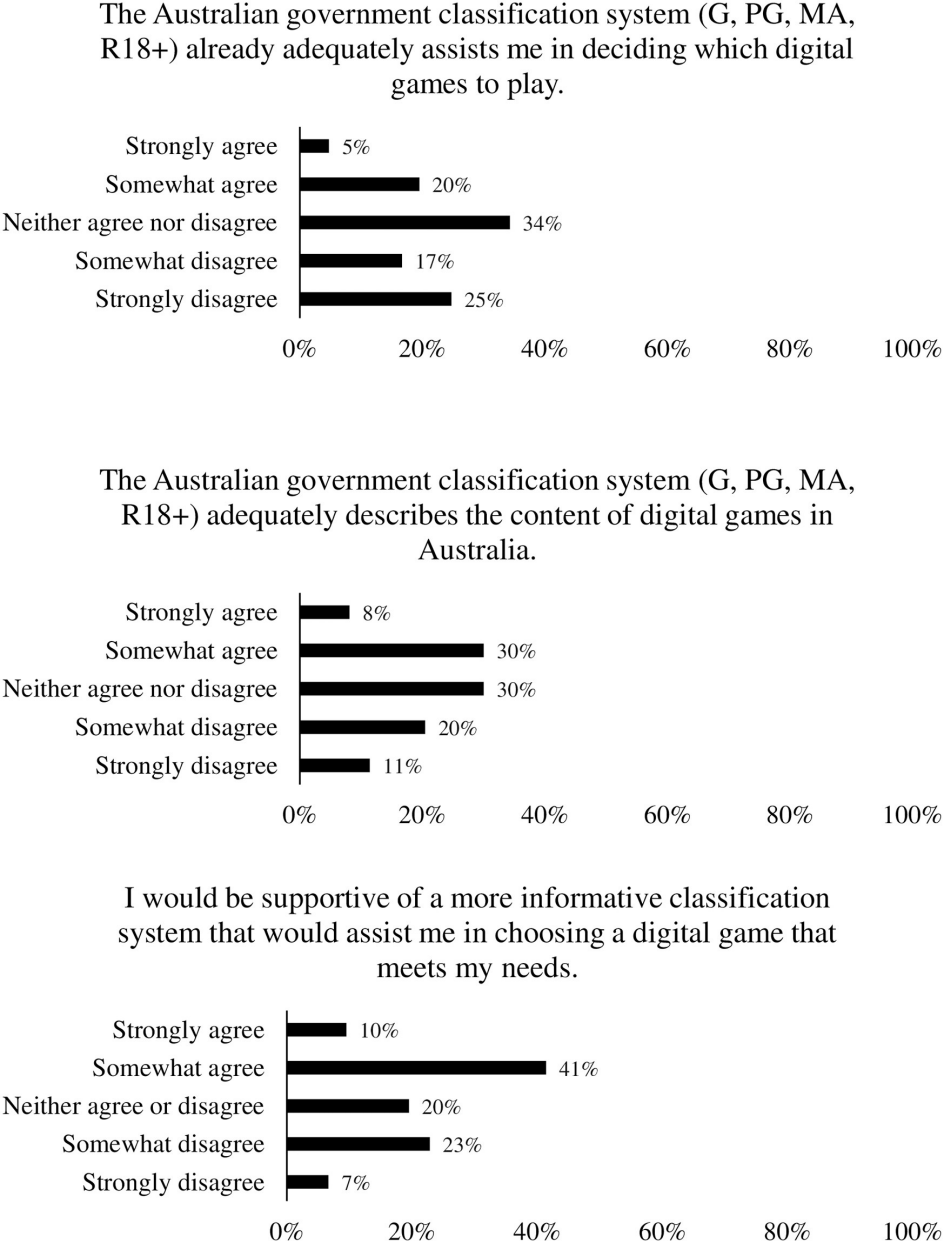
Response distributions for attitudes towards current and potential game classification systems.

Attitudes towards the current classification system and potential new classification systems have been reported in Fig 6. Importantly, almost half the participants disagreed that the current classification system already adequately assists them when choosing a game. Furthermore, around half of participants agreed that they would be supportive of a more informative system.

### Qualitative analysis of attitudes toward classification systems

Participants were asked to write a free text response to the following question:

“Can you explain your attitude toward classification systems when purchasing games?”

The responses to this question were analysed using a conventional approach to qualitative content analysis, with preconceived categories being avoided, and instead being determined by the implementation of the coding process [see e.g. 31]. As a result of this analysis, four categories emerged from the data (two of which represented positive attitudes towards the current Australian National Classification Scheme for Computer Games and two of which represented negative attitudes), and we now discuss each of these emergent categories in the context of the participants’ responses.

#### 1. They are needed for children

This category relates to those participants who noted that the classification system was necessary in order to assist adults when buying video games for under 18s. This was by far the most common category to emerge in terms of why participants chose to engage with the current Australian video game classification system. Typical comments included:

“I only look at classifications when buying/looking for a gift for a child.”

And

“I follow it very strictly for my children.”

For the most part, participants in this category agreed that the Australian National Classification Scheme was a good idea, and that it served its purpose, but several participants did not think that it was entirely suitable, either because it was over restrictive, or else did not go far enough. The gaming literacy of the participants themselves likely played an important role in their use of the classification system, with regular gamers more likely to use the classifications as the start of a dialogue, rather than as a strict barrier for exclusion, for example:

“If my children want to play a game I’m unfamiliar with, I will use the rating and watch YouTube videos to help make a decision.”

And

“My eldest son is a gamer with more experience with games than myself. For him I allow him to make his own choices rather and just keep communication open.”

These findings are consistent with the work of Nikken, Jansz [32], who found that parents’ attitudes towards video game classification systems are associated with the parents’ own gaming and views on positive game effects.

#### 2. They help to identify adult themes

Of the participants who found aspects of the current Australian National Classification Scheme to be useful, a second (weaker) category to emerge was that they helped participants to identify “adult themes” that they found to be distasteful in video games. These were largely those games that involved violence, drugs, and scenes of a sexual nature, for example:

“They’re useful in deciding if it’s the right game for me. I don’t like to see a lot of violence or nudity in my games so it can be helpful that way.”

Again, in many of these instances the participants found the classifications to be a useful starting point for them to determine if they wanted to play a game or not, rather than a hard rule to stick by:

“If R or MA, I might try and analyse them to determine if this game is right for me, (e.g., if the violence is gratuitous or not).”

#### 3. They are irrelevant for adults

This category is the most strongly emergent in terms of the partial redundancy of the current Australian National Classification Scheme, with participants noting that once they reached adulthood, they paid very little attention to the classification system. For example:

“Irrelevant. I am mature enough to make my own decisions.”

And

“I’m over 18 so don’t care about ratings. Don’t influence my decision.”

These responses highlight the need for a classification system that goes beyond simply stating the extent to which games are or are not age appropriate. In some instances, the current classification system even acted to put off mature gamers from games that received a lower ager ranking, for example:

“Games with a lower classification aren’t necessarily bad, I certainly play games designed for younger people, but I know that the people I may purchase for would prefer something they would consider more age appropriate’”.

This category is in opposition to the second category to emerge from the participants’ responses, indicating that whilst some adults would value a classification system to help them identify adult themes, others find such a system to be redundant. These results would point to the need for a classification system that went beyond simply identifying if a video game was “age appropriate” or not, as for many individuals this is not simply a question of age.

#### 4. They are arbitrary and inaccurate

The second main category to emerge from the data in terms of why the participants found the current Australian National Classification Scheme to be redundant is because they found it to be either arbitrary or inaccurate. The following two comments are representative of this category, and highlight why participants found the current classification system to be ill-serving:

“Violence and implied sexual relationships are rated the same where they aren’t equally ‘difficult’ for younger people to understand.”

And

“The Australian system is outdated and flawed. It harshly penalises video games that contain drug use but accepts rape and torture.”

This lack of nuance regarding the classification of media texts has long been argued by media scholars [33] and has been noted as being particularly problematic because of the subjective and affective nature of game play [34].

## Discussion

The purpose of the present study was to collect data to better understand the digital game selection process and determine what role a current national classification system plays in this process. In addressing our first research question (what are the leading strategies used by players to make game decisions?), we can surmise that players largely turn to communities of play (from friends to Twitch streams) and reviewers for guidance on whether a game will suit them. In regard to our second research question (do players positively or negatively appraise the game selection process?), while we find evidence that the game selection process is largely a positive experience, the adoption of varied selection strategies can be onerous and complicated to navigate, as supported by the 40% of participants who agreed that choosing a game is time consuming. Third, (do players regard the game selection process as difficult?), 50% of our respondents agreed that it was difficult to find a game to suit their needs. Fourth, (what are players’ attitudes towards the Australian National Classification Scheme?), the Australian National Classification Scheme is largely disregarded in the game selection process. Finally and importantly, for our fifth research question (would players be supportive of a more informative classification system?) there was general support for a more informative classification system to help consumers understand game content and make appropriate game selection decisions.

As can be seen from the emergent categories in the qualitative analysis, the responses largely aligned with those of the quantitative responses shown in Fig 5; that is, it appears that the current Australian video game classifications are largely disregarded by participants who were choosing a game for themselves but useful for those who were choosing a game for a child or someone that they did not considered to be a “reasonable adult”. These qualitative responses reveal some further nuances into this delineation, and in particular for the participants in this study it would appear that those who choose not to use the classification system do so either because they do not think it is appropriate for them as adults or because they believe it to be inadequate. Similarly, for those who do use the classification system (i.e., mainly those people buying video games for the under 18s), it is clear that the current classification system falls well short of providing all of the information that is needed to help encourage dialogue about the suitability of games for the intended audience.

The lack of perceived utility in game ratings systems can be explained by a number of confounding factors in terms of their use. First, as Flew explains, the rise of digital media has brought many complications to the way that classification systems are developed and operate [12]. The Internet facilitates fast, decentralised content production, distribution, and consumption through many content producers [12]. As a result it is recognised that the vast majority of online content will never be formally classified, with the responsibility for censorship essentially falling to service providers (such as Apple in the case of the App Store, and Google in the case of YouTube) [11]. This means that ratings, classification and censorship often take place without any public oversight or awareness. This lack of transparency is a product of both “platformisation” and consumer expectations. While the Australian Classification Board does have a publicly accessible database of information that provides more explanation of their ratings decisions, presenting this information as part of packaging is not industry practice and so there is very little consumer knowledge about its existence.

Second, the definition of “community expectations” used for ratings purposes is contested and not necessarily respected by game players [12]. Classification systems are subject to review to align with such expectations and the Australian Classification website contains numerous community research reports that demonstrate engagement with community expectations [35, 36]. However the difficulty in defining what an adult finds reasonable may result in the perception that certain media content is unfairly classified [17], and at times governments may fail to quickly align with shifting public interests [37].

Third, as digital platforms such as YouTube are global, “there is a non-correspondence between their geographical space of activity and national territorial jurisdictions”. To ease the classification process for developers seeking to release content worldwide, the Australian board is encouraged to match up with other nations, and work with content and service providers [11, 14]. In 2017, the Australian Government approved the use of the International Age Rating Coalition tool, an international classification system, for mobile games. To use the tool, a game developer answers a questionnaire that generates an Australian rating [38]. Fourth, it is now less clear what counts as “media content”, and what counts as “personal communication”, and how those two types of media experience can be regulated simultaneously [12]. In light of these issues it is clear that the National Classification Scheme must be continually developed to be useful in the digital age to remain relevant to the public.

One of the key implications of this research is that national classification systems only service a fraction of consumers (parents and children), with the remaining market needing to adopt varied strategies to make game selection decisions. Indeed, a recent report by Bond University as part of their ongoing “Digital Australia” research indicates that 78% of Australian players are aged 18 years or older with an average age of 34 years [39]. Accordingly, there is currently unprecedented demand for commercially unbiased classifications of games which extends beyond “what pleasures, knowledge and experiences are deemed appropriate for minors” [17], and protecting individuals from material they find offensive [18]. Because National Classification Systems are essentially funded by the tax payer, they offer a unique opportunity to provide unbiased and standardised evaluations of games which could be broadly valuable to the digital game consumer. That is, they *could* represent an attractive alternative to more complex, time consuming, and untrustworthy sources. However, our results show that, in their current form, National Classification Systems are not currently attractive to adult players when choosing games for themselves.

We have already articulated several reasons why National Classification Systems are limited in their scope for addressing the needs of players more broadly, including the subcontracting of censorship to producers and distributors (e.g., Apple, Google) [11, 12], ambiguity regarding the definition of a “reasonable adult” [12, 15], increasing need for globalised and transparent classification standards [11, 14], and ambiguity regarding what counts as media content [12]. The resolution of these issues represents a complex task which would involve considerable resources. However, given the size of the video game market, the revenue it produces, and the number of stakeholders involved, there is a strong impetus to further research and address these issues. A well-defined, transparent and internationalised classification scheme would allow developers to design their games more strategically, distributors to promote games more accurately, and players to find suitable games more easily.

### Limitations

While we believe our study makes a useful contribution towards justifying the development of more informative digital game classification systems, we acknowledge that our methodology had several limitations. Firstly, we relied on a self-selected sample recruited via online survey methods. As a result the quality of the responses may have been compromised by the anonymity and uncontrolled nature of the data collection. Similarly because our survey was initially distributed through Australian networks, our data is likely to be skewed by a predominantly Australian selection of respondents, who were asked to answer questions about the Australian Classification System. We can therefore only make conclusions regarding attitudes towards the Australian Classification System and recommend that similar research be conducted in other regions to understand whether similar attitudes exist for National Classification Systems broadly, or if they are unique to the Australian System. Finally, we only secured a relatively small subsample of adults who buy for children and did not seek responses from those under the age of 18. Given that the utility of the Australian Classification system appears to largely relevant to those with age considerations when choosing digital games, further research is needed to determine the value of such systems in younger age groups and for parents/guardians.

However, we believe that the mixed method approach to our research has enabled us to negotiate these limitations and produce results that we suspect are replicable in studies of the utility of other classification systems, and therefore have enhanced the understanding of the benefits and limits of a National Classification System in general. We encourage future researchers to utilize similar approaches when exploring the role of National Classification Systems on consumer decisions.

## Conclusions

Overall, these results would seem to suggest a more informative classification system would improve the process of choosing games for the majority of consumers. While there seems to be good awareness of the value of the existing classification system for parents (and others) who are choosing games for children, it is equally clear that the existing classification system is not used to help guide game choice for most adults. Given the subjective but powerful nature of gameplay experiences, we would suggest any future classification system for games should not only highlight the severity and occurrence of adult themes in video games but should also use data from reviews and communities of play to help adults more readily identify the right game for them. As video games become increasingly prevalent and internationalised there is a clear imperative to create an improved system for relating the affective content of games in a sophisticated, nuanced and universally respected format. Ultimately the challenge lies in developing a classification system which adequately describes game content, whilst efficiently delivering this information to players in a standardised and unbiased format.

## Supporting information

S1 File Why don’t we play games?. (DOCX)

S1 Data Why don’t we play games?. (XLSX)
