# Supplementary material for: Evidence that digital game players neglect age classification systems when deciding which games to play
Source: PLoS One. 2022 Feb 22;17(2):e0263560. doi: 10.1371/journal.pone.0263560 (PMC8863231; doi:10.1371/journal.pone.0263560)
Supplement: S1 File — (DOCX) [file pone.0263560.s001.docx]

Why don't we play games?

Start of Block: Intro

Thank you for taking part in this survey. The following information outlines a research project being conducted by a team of researchers from four universities in Perth, Western Australia.


This is a short survey to help us better understand what factors influence people's decisions to play games. By games we mean computer games, mobile games, board games, sports - any form of play that involves rules. A lot of questions relate to digital games, but you'll only be shown these questions if you play those games. By digital games we mean digital, video, or computer games - any game that is played on a digital device. Regardless of whether you play digital games or not, we would still really appreciate your response.


You have been invited to participate and your involvement is completely voluntary. You may withdraw from the survey at any time. To do so, close the browser, and all incomplete responses will be removed. The questionnaire will require approximately 10 minutes to complete. If you agree to participate in the survey, please complete the questions that follow. Your responses will be anonymised and will not be used individually. If any potentially identifiable information is provided during open-ended questions, it will be anonymised.


At the end of the survey you will be invited to fill out contact details so you can go into the draw for one of three $50 JB Hi-Fi vouchers. This information is only used to contact the winners of the draw, It is stored securely and is in no way associated with your survey responses.


Please be aware that a member of the research team might be a lecturer in a course that you are completing, but your participation, or non-participation, will have no bearing on the relationship with this lecturer.
There are no risks associated with conducting this survey, but if you are unclear about anything, or if you want to have your responses removed at any time then please contact Dr Tauel Harper at tauel.harper@uwa.edu.au.

Approval to conduct this research has been provided by the University of Western Australia, in accordance with its ethics review and approval procedures. Any person considering participation in this research project, or agreeing to participate, may raise any questions or issues with the researchers at any time, by contacting Dr Tauel Harper at tauel.harper@uwa.edu.au.

In addition, any person not satisfied with the response of researchers may raise ethics issues or concerns, and may make any complaints about this research project by contacting the Human Ethics Office at the University of Western Australia on (08) 6488 3703 or by emailing to humanethics@uwa.edu.au

Q39 I have read the above statement and understand that my answers are anonymous and I retain the right to withraw them from the study at any time.

- Yes (7)
- No (8)

Skip To: End of Survey If I have read the above statement and understand that my answers are anonymous and I retain the rig... = No

End of Block: Intro

Start of Block: Do you like games?

Q1 Do you like to play games?

- Yes (1)
- No (2)

End of Block: Do you like games?

Start of Block: General discouragement

Q2 If you could name one thing that stops you from playing a game, what would it be?

________________________________________________________________

End of Block: General discouragement

Start of Block: General encouragement

Q3 Can you name one thing that playing a game would need to offer to encourage you to play it?

________________________________________________________________

End of Block: General encouragement

Start of Block: Relative concerns

| 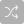 | 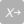 |
| --- | --- |

Game Play Factors How concerned are you about the following factors when deciding whether or not to play a game?

|  | Not at all important (1) | Slightly important (2) | Moderately important (3) | Very important (4) | Extremely important (5) |
| --- | --- | --- | --- | --- | --- |
| Effect on physical health (1) |  |  |  |  |  |
| Effect on mental health (2) |  |  |  |  |  |
| Degree of social interaction (3) |  |  |  |  |  |
| Treatment of diversity (4) |  |  |  |  |  |
| Engagement with social and political issues (5) |  |  |  |  |  |
| Replayability (6) |  |  |  |  |  |
| Game length (7) |  |  |  |  |  |
| Challenge level (8) |  |  |  |  |  |
| Value for money (9) |  |  |  |  |  |

Explanation If you would like to add more explanation about your selections in this table you can do so here

________________________________________________________________

________________________________________________________________

________________________________________________________________

________________________________________________________________

________________________________________________________________

Other factors
Are there any other factors that influence your decision to play (or not to play)?

________________________________________________________________

End of Block: Relative concerns

Start of Block: social discouragement

Discouraging Attribu What sort of game features or attributes do you think generally discourage people from playing games?

________________________________________________________________

________________________________________________________________

________________________________________________________________

________________________________________________________________

________________________________________________________________

End of Block: social discouragement

Start of Block: Information about gaming habits

Frequency Play How often do you play digital games?

- Daily (1)
- 2-3 times a week (3)
- Once a week (4)
- Monthly (6)
- Less than once a month (7)
- Never (5)

Display This Question:

If How often do you play digital games? = Never

Why don't play digit Why don't you play digital games?

________________________________________________________________

________________________________________________________________

________________________________________________________________

________________________________________________________________

________________________________________________________________

Display This Question:

If How often do you play digital games? != Never

Play quantity On average, how many hours per week would you play digital games?

- less than one hour (1)
- 1 -3 hours (2)
- 3-6 hours (3)
- 7-10 hours (4)
- 11-15 hours (6)
- 16-20 hours (7)
- more than 20 hours (5)

Display This Question:

If How often do you play digital games? != Never

Acquisition
How do you generally acquire digital games?
 
*Please select all which apply*

- purchase via a mobile phone or tablet app store (1)
- free download via a mobile phone or tablet app store (10)
- purchase hard copy from a bricks-and-mortar retail outlet (2)
- purchase digital copy from a bricks-and-mortar retail outlet (8)
- purchase hard copy from an online retail outlet (a website) (7)
- purchase a digital copy from an online retail outlet (a website) (9)
- purchase digital download (such as Steam, Itch, PlayStation Network, etc., to a PC or console) (3)
- free digital download (such as Steam, Itch, PlayStation Network, etc., to a PC or console) (11)
- sharing (with friends and family) (4)
- peer to peer sharing/torrenting sites (5)
- streaming online (6)

Display This Question:

If How often do you play digital games? != Never

Platforms On what platforms do you usually play games?

- Personal computer/laptop (1)
- Playstation (2)
- Xbox (3)
- Nintendo Wii (4)
- Nintendo Switch (5)
- mobile phone/tablet (6)
- Nintendo DS (7)
- Playstation PSP (8)
- other (9) ________________________________________________

Display This Question:

If Do you like to play games? = Yes

Games played In the last three months which games have you played the most?

- most played (1) ________________________________________________
- second most played (2) ________________________________________________
- third most played (3) ________________________________________________

Display This Question:

If Do you like to play games? = Yes

Other players? Who else in your household plays games?

- Just me (1)
- My partner (2)
- My child/children (3)
- Other people (family, friends, housemates etc.) (4)

End of Block: Information about gaming habits

Start of Block: Purchasing decisions

Purchase games? Do you purchase digital games for yourself or others?

- I purchase for myself only (1)
- I purchase for others only (2)
- I purchase for both myself and others (4)
- I never purchase digital games (3)

Display This Question:

If Do you purchase digital games for yourself or others? != I never purchase digital games

Annual spend
Please estimate how much money you spend on digital games annually
 
*Please include the annual costs of any subscriptions that you may have to game services (e.g., Xbox game pass), loot boxes, microtransactions, etc.*

- $0 (2)
- $1-$49 (8)
- $50-$99 (3)
- $100-$199 (4)
- $200-$499 (5)
- $500-$999 (6)
- more than $1000 (7)

Display This Question:

If Do you purchase digital games for yourself or others? = I purchase for myself only

attention ratings Do you pay attention to classifications (such as R, MA, PG) when choosing a game to purchase for yourself?

- Never (1)
- Sometimes (2)
- About half the time (3)
- more than half the time (4)
- Always (5)

Display This Question:

If Do you purchase digital games for yourself or others? = I purchase for both myself and others

attention Do you pay attention to classifications (such as R, MA, PG) when choosing a game for the following people?

|  | Never (1) | Sometimes (2) | About half the time (3) | More than half the time (4) | Always (5) | N/A (6) |
| --- | --- | --- | --- | --- | --- | --- |
| Yourself (1) |  |  |  |  |  |  |
| A friend or partner (6) |  |  |  |  |  |  |
| A child (under 16) (2) |  |  |  |  |  |  |
| Other (8) |  |  |  |  |  |  |

Display This Question:

If Do you purchase digital games for yourself or others? = I purchase for others only

attention others Do you pay attention to classifications (such as R, MA, PG) when choosing a game for the following people?

|  | Never (1) | Sometimes (2) | About half the time (3) | More than half the time (4) | Always (5) |
| --- | --- | --- | --- | --- | --- |
| A friend or partner (1) |  |  |  |  |  |
| A child (under 16) (2) |  |  |  |  |  |
| other (4) |  |  |  |  |  |

Display This Question:

If Do you purchase digital games for yourself or others? != I never purchase digital games

ratings attitude Can you explain your attitude toward classification systems when purchasing games?

________________________________________________________________

________________________________________________________________

________________________________________________________________

________________________________________________________________

________________________________________________________________

Display This Question:

If Do you purchase digital games for yourself or others? != I never purchase digital games

Q21 Do you engage with game reviews when choosing a game?

- Always (1)
- More than half the time (2)
- About half the time (3)
- Sometimes (4)
- Never (5)

Display This Question:

If How often do you play digital games? != Never

| 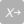 |
| --- |

game choice When choosing a digital game to play, which of the following do you use to help you make a decision?

|  | Never (1) | Sometimes (2) | About half the time (3) | Most of the time (4) | Always (5) |
| --- | --- | --- | --- | --- | --- |
| Australian Classification System (e.g., R, MA, PG) (1) |  |  |  |  |  |
| Game Reviews (videos or articles) (2) |  |  |  |  |  |
| Social Media (7) |  |  |  |  |  |
| Recommendations from people you know (3) |  |  |  |  |  |
| Game cover art (4) |  |  |  |  |  |
| Game trailer (5) |  |  |  |  |  |
| Other (6) |  |  |  |  |  |

Display This Question:

If How often do you play digital games? != Never

And Do you purchase digital games for yourself or others? = I purchase for myself only

Or Do you purchase digital games for yourself or others? = I purchase for both myself and others

| 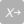 |
| --- |

experience -self How would you describe the experience of choosing a digital game that ***meets your needs***?

|  | Strongly disagree (1) | Somewhat disagree (2) | Neither agree nor disagree (3) | Somewhat agree (4) | Strongly agree (5) |
| --- | --- | --- | --- | --- | --- |
| Enjoyable (3) |  |  |  |  |  |
| Time consuming (1) |  |  |  |  |  |
| Frustrating (2) |  |  |  |  |  |
| Confusing (4) |  |  |  |  |  |
| Boring (8) |  |  |  |  |  |
| Satisfying (9) |  |  |  |  |  |

| Page Break |  |
| --- | --- |

Display This Question:

If Do you purchase digital games for yourself or others? = I purchase for others only

Or Do you purchase digital games for yourself or others? = I purchase for both myself and others

| 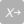 |
| --- |

experience others How would you describe the experience of choosing a digital game that ***meets the needs of others***?

|  | Strongly disagree (1) | Somewhat disagree (2) | Neither agree nor disagree (3) | Somewhat agree (4) | Strongly agree (5) |
| --- | --- | --- | --- | --- | --- |
| Enjoyable (3) |  |  |  |  |  |
| Time consuming (1) |  |  |  |  |  |
| Frustrating (2) |  |  |  |  |  |
| Confusing (4) |  |  |  |  |  |
| Boring (8) |  |  |  |  |  |
| Satisfying (9) |  |  |  |  |  |

| Page Break |  |
| --- | --- |

Display This Question:

If Do you purchase digital games for yourself or others? != I never purchase digital games

Why reviews What influences your decision about whether to pay attention to reviews and ratings when choosing a game?

________________________________________________________________

________________________________________________________________

________________________________________________________________

________________________________________________________________

________________________________________________________________

| Page Break |  |
| --- | --- |

End of Block: Purchasing decisions

Start of Block: Sentiment analysis

Q23 In this section please indicate how much you agree with each statement.

Display This Question:

If How often do you play digital games? != Never

| 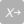 |
| --- |

Difficult Before playing a digital game, it is often difficult to determine whether a game will meet my needs.

- Strongly agree (1)
- Somewhat agree (2)
- Neither agree or disagree (3)
- Somewhat disagree (4)
- Strongly disagree (5)

| 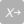 |
| --- |

Supportive I would be supportive of a more informative classification system that would assist me in choosing a digital game that meets my needs.

- Strongly agree (1)
- Somewhat agree (2)
- Neither agree nor disagree (3)
- Somewhat disagree (4)
- Strongly disagree (5)

| 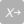 |
| --- |

Save time I would be supportive of a more informative classification system that would save me time when choosing a digital game that meets my needs.

- Strongly agree (1)
- Somewhat agree (2)
- Neither agree nor disagree (3)
- Somewhat disagree (4)
- Strongly disagree (5)

Display This Question:

If How often do you play digital games? != Never

| 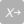 |
| --- |

Describes content The Australian government classification system (G, PG, MA, R18+) adequately describes the content of digital games in Australia.

- Strongly agree (1)
- Somewhat agree (2)
- Neither agree nor disagree (3)
- Somewhat disagree (4)
- Strongly disagree (5)

Display This Question:

If How often do you play digital games? != Never

| 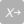 |
| --- |

Assists decision The Australian government classification system (G, PG, MA, R18+) already adequately assists me in deciding which digital games to play.

- Strongly agree (1)
- Somewhat agree (2)
- Neither agree nor disagree (3)
- Somewhat disagree (4)
- Strongly disagree (5)

End of Block: Sentiment analysis

Start of Block: Demographics Base/Universal

demographic Some information about your personal details will help us produce more insightful research. We would like to reiterate that information provided in this survey is completely anonymous.

age
How old are you?

▼ under 16 (1) ... 70 or older (12)

edu What is the highest level of school you have completed or the highest degree you have received?

- Less than high school degree (1)
- High school graduate (2)
- Technical and vocational training certification (4)
- Some university but no degree (3)
- Bachelor's degree (5)
- Master's degree (6)
- Doctoral degree (7)
- Professional degree (JD, MD) (8)

race With which racial and ethnic group(s) do you identify?

- Australian (4)
- Indigenous Australian or Torres Strait Islander (5)
- New Zealander or Pacific Islander (6)
- Asian (7)
- Indian (8)
- Middle Eastern (9)
- British or European (10)
- North American (11)
- South American (12)
- African (13)
- Other (please specify) (15) ________________________________________________
- ⊗Prefer not to answer (16)

| Page Break |  |
| --- | --- |

gender How would you describe your gender?

- Female (3)
- Male (4)
- Other (please specify) (5) ________________________________________________
- Prefer not to answer (6)

sexual id How would you describe your sexual identity?

- Heterosexual (4)
- Homosexual (5)
- Bisexual (6)
- Other (please specify) (7) ________________________________________________
- Prefer not to answer (8)

| Page Break |  |
| --- | --- |

Parent Which of the following best applies to you?

- I am not a parent/guardian (1)
- I am a parent/guardian currently responsible for child(ren) some or all of the time (3)
- I am a parent/guardian not currently responsible for child(ren) (e.g., they have left home) (4)
- Other (5) ________________________________________________

| Page Break |  |
| --- | --- |

Display This Question:

If Which of the following best applies to you? = I am a parent/guardian currently responsible for child(ren) some or all of the time

Children age
Please select all which apply


As a parent/guardian, I am responsible for children...

- under the age of 5 years (1)
- aged between 5-10 years (2)
- aged between 11-14 years (3)
- aged between 15-17 years (4)
- aged 18 years or over (5)

| Page Break |  |
| --- | --- |

health Do you have health problems that impact your use of, or engagement with, digital games?

- Yes (please provide details) (1) ________________________________________________
- No (2)

income Please indicate the answer that includes your entire household income in 2018-2019 before taxes.

- Less than $20,000 (1)
- $20000 to $39,999 (2)
- $40,000 to $59,999 (3)
- $60,000 to $79,999 (4)
- $80,000 to $99,999 (5)
- $100,000 to $119,999 (6)
- $120,000 to $159,999 (7)
- $160,000 to $199,999 (9)
- $200,000 to $249,999 (11)
- more than $250,000 (13)

| 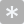 |
| --- |

postcode What is your post code?

________________________________________________________________

Q49
Thank you! [Click here](https://uwa.qualtrics.com/jfe/form/SV_6ExZ4r2TrhVDMjz) to enter the draw to win one of three $50 JB Hi-Fi vouchers.
 
(This will take you through to a separate form to ensure that no personal information is associated with this survey response)

End of Block: Demographics Base/Universal
